# “Report on the Medical College” Again

**Published:** 1842-10

**Authors:** 


### "REPORT ON THE MEDICAL COLLEGE" AGAIN.

In our last number we noticed briefly a "Report" made to the City Council on the subject of the Medical College, in which the conduct and motives of the Faculty were assailed, and which, after having been rejected by the Council, was published and sent abroad over the country. We stated at the same time that the Faculty were preparing a reply to the unfounded statements and unjust imputations contained in this document. That reply is before us now, in a pamphlet setting forth the "origin and present condition of the Louisville Medical Institute." It is a plain, unvarnished statement of facts, temperate in its tone—very temperate indeed under all the circumstances. As copies of it have been sent to the patrons of the Journal, we think it unnecessary to indulge in copious extracts from it, as was our intention, and shall therefore only advert briefly to one or two points connected with the matter.

The Dean, in the pamphlet before us, dives at once to the source of this movement, and of course looks beyond the Committee. He says:

"It is known to the citizens of Louisville that there is a party in the city who have for some years been endeavoring to produce discontent with the management of the Medical Institute of Louisville. The Managers and Professors have borne their repeated expressions of dissatisfaction, publicly and privately made, and their attempts to take the school out of their hands, with quite as much patience as is commonly shown in similar circumstances. Some indeed have manifested a natural and justifiable indignation at these unworthy efforts and schemes; but they have avoided a public expression of it, and no public defence has yet been made. They have felt extreme reluctance to a controversy with men who appeared to be proceeding from outrage to outrage, in hope of provoking one, from which they hoped to gain notoriety at least, and in which they had little to lose. But inasmuch as these men have at length succeeded in misleading some of the Council so far as to have their views presented to that body in the shape of a report, which has thus acquired some appearance of importance; although the report was rejected, it has been thought advisable by the Faculty, to place the matter in its true light before the friends of the school, some of whom may be misled into believing the statements made in the report.

Indeed the temper of the rejected report and the character of its statements are such as to lead the Faculty to the conviction that the enmity which prompts these movements is not to be mitigated by time nor softened by forbearance; and some extraordinary acts of those who can no longer be considered in any other light than that of determined enemies of the school as now constituted, as well as the inquiry of a number of citizens, have led them at length to the opinion that it is time to check the influence which has misled some of the Council, and which left unnoticed might mislead others.

These men indeed sometimes deny that they have any intention, in these efforts to obtain possession of the management of the Medical School, to disturb the present professors. But it is not to be believed that, if after years of intrigue they could by any possibility get the administration of the school into their hands, they would cease until they attain their main object, openly avowed two years ago, the possession of the chairs. It is not to be believed that men who have arranged a plan for delivering lectures, and have asked for the use of the Medical Institute for the purpose, would fail to take possession of it if they should dispossess the Managers whose sense of duty and propriety compelled them to deny them. It is evident that the object was, by succeeding in carrying the report, which they without foundation supposed would give them possession of the Institute, to do that which they openly proposed to do in 1840, eject the present Professors.

Such are the motives which led to the statements in the rejected report, and to the improper use that has since been made of it. * * * And so completely have the members of the committee entered into the feelings of the inimical party, that, according to the statement of the editor of the Gazette, which has not been contradicted, they had a great number of copies of the report printed, with the ayes and noes attached, in a form evidently intended for distribution; and one of the committee has stated to a gentleman, although he was told that the information was sought for in order to be used, that the publication was made in consultation* with Dr. Bell, Dr. Bullitt, and Dr. Flint, and from the interest they manifested in it he supposed they would be willing to pay a part of the expense if called on."

As it respects the transfer of the Square, buildings, &c., from the occupancy of the Medical Institute, which was the ostensible object of the committee, the report, so much of it as is devoted to that object, proceeds on the assumption that the Louisville College is the corporate body alluded to in the deed of trust by which the Managers of the Institute at present hold the property. Now the rejoinder of the Faculty makes it about as plain as words can make it, that this College and its trustees were no more in the minds of the City Council or of the people at their public meeting than an Indian wigwam and its inmates!—not a bit more! No reference was made to them in any way, nor can the language of the indenture or the resolutions of the citizens, by any legitimate philological torture, be made to refer to them. And yet this "elaborate report", whose statements, according to the editor of the Louisville Gazette, are "undoubtedly authentic", says, "nobody

* The member of the committee here referred to, has since stated in one of the city prints, that the expression "made in consultation with," &c., is a misapprehension.—Ed. Jour.

thinks nor has thought of establishing any other school in Louisville!" The following extract from the exposition of the Faculty contains a summary of the evidence offered by them on this point, and shows on what grounds the transfer is to be made.

"The proposition to request a transfer of the management of the school from the present Board, rests on the ground of an asserted right arising from the donation of the Council.

The right of management was in the Board of Managers of the Medical Institute of Louisville years before the donation was made. It was the knowledge of the powers they possessed which led the Council to make the donation, and was the avowed ground on which it was made. The donation therefore gives no right of management to the Council.

What then are the legitimate claims of the Council upon the school? They are those only which arise out of the terms of the donation when accepted: for it is the acceptance of a donation which subjects the receiver to the restrictions contained in the terms of the gift. Beyond these the claim of the donor does not extend.

In the present case the terms of the donation which was accepted by the Managers were these, beyond which the Council have no claim. 1. That the property should be used by the Managers for the purpose of carrying into effect their previously possessed powers of establishing a medical school: and, 2dly, that in the event of the Council's carrying the views of the citizens fully into effect, by erecting other college buildings on University Square, and obtaining a charter for a university, the Managers are to transfer, if the Council request it, the square, buildings, &c., to the trustees of the university.

The first authorises them in case the property is used for any other purpose than that of a medical school, to reclaim possession of it. But as long as it is used for the purpose for which it was given, they cannot move in the matter. How far a great failure notwithstanding the efforts of the Managers to raise up a school sufficient to satisfy the expectations of the donors, might authorise the latter to interfere, need not be considered; seeing that in the present case the utmost expectations of the whole city have been far exceeded.

The second authorises the Council to build other college buildings on University Square, and having obtained a charter, to require the Managers of the Medical Institute to transfer the square, &c., to the trustees named in it. Before this is done there is no ground for the request.

In case however that the wishes of the citizens expressed in their meeting in 1837 should be carried into effect and buildings be erected for the other departments, and the managers of the Medical Institute should be called on to reconvey the square, &c., it is the property only that is to be reconveyed, by right of the reservation in the deed of donation; which can go no farther than to call back what was granted; viz. the property, not the right of management, which was in the Board of Managers before the donation was made.

When that time shall arrive it will be a matter of consideration on what terms the Medical Institute of Louisville, called by anticipation the medical department of the intended university, shall really become so. These will no doubt be such as shall be agreed to by those under whose charge it has increased beyond all expectation and beyond all precedent, and in all probability will continue to increase."

One of the most calumnious charges made against the Faculty by the authors of the rejected report, is that of having embezzled the funds of the institution. But a small portion, they say, of the monies derived from the matriculation fees have been devoted to the purposes of the school, "the residue having gone with all the other revenue of the establishment into the Professors' pockets." So far from this being the case, it appears from a detailed statement of the expenditures given in the pamphlet, that "the expenses of the School have exceeded by several thousand dollars the amount of matriculation fees received;" in fact, that the Faculty have taken money out of their pockets to the amount of $8,000 over and above that fund.

But perhaps the most perfectly gratuitous suggestion contained in the report, and at the same time the one most ruinous and destructive to the school, is that the proceedings of the Board of Managers have been to such a degree informal "as to vitiate and nullify in a legal sense all their recent acts"; and of consequence, that "the appointment of professors, the financial arrangements of the concern, the distribution of collegiate advantages, and the conferring of degrees, are done without authority of law, and of course are invalid and worthless to all interested." "It appears," says the report, "that there are still living three of the original corporators of the Medical Institute, who have neither participated, nor been summoned to participate in any of its transactions for the last four or five years—a portion of the associates having proceeded to exercise all the corporate functions, without any regard to the equal rights of the excluded members."

To this most injurious statement, which strikes directly at the very existence of the institution, and yet is made under the garb of the most disinterested friendship, the Dean, after directing attention to the early history of the Institute as previously given by him in the pamphlet, and which shows the utter groundlessness of the assertion, replies more particularly as follows:

"The three surviving *members* here spoken of, are medical men, who nine years ago excluded themselves from all participation in the management of the Institute, by passing the regulation that no medical man should thereafter act as manager. This, being managers at the time, they had full authority to do by the act of incorporation in 1833; and the Legislature confirmed it by the act of 1835 as the permanent law of the school.

'The portion of the associates (spoken of) who proceeded to exercise all the corporate functions,' are four still surviving managers of that Board, which the old three abovementioned (and their then associates) constituted nine years ago; together with those who have been since elected. In electing new Managers instead of those whose places were vacated by death, resignation or removal, they were debarred from choosing any of the surviving three medical men, by the law of the Institute made by these three themselves: and were compelled to appoint the same men managers because there were no others eligible, as the three had themselves arranged the matter when they brought in the non-medical men expressly to be made Managers.

If the three had been present at elections they could have carried no candidate, as they were from the beginning in the minority, six members, medical men, having chosen nine men not medical, and made them managers, If they had even had a majority it would have made no difference; because there were no others to choose than the non-medical men who constituted the old Board. Neither would a majority have enabled them to bring in others to make managers of; because the Board had the right by the original act of 1833 to determine the mode and manner of admitting new members, and how they shall cease to be such; and no one could be admitted unless nominated by the President or Moderator. By the operation therefore of the original act of incorporation in 1833, and the regulation made by the three medical men, the result was the same whether there was an election or not; viz. the perpetuation of the office in the hands of the Board."

The rejected report contains other accusations and complaints, which do not involve perhaps any direct criminality on the part of the Faculty, but still are of a kind to excite prejudice in the minds of the uninformed. These we shall not notice further than to say that they are fully refuted by the pamphlet, and shown to be as destitute of foundation and as little worthy of confidence as those to which we have adverted.

That the committee should have preferred the grave charges contained in this paper confessedly without "authentic information," is certainly most extraordinary, and comports badly with their claim to sincerity. And it is equally extraordinary that, after a majority of their fellow council-men had cast the thing of shreds and patches from them as though it were something unclean, they should proceed to have it printed, and threaten the Faculty with its distribution if an attempt were made to discredit it! Would it not seem, indeed, that they feared the exposure which has followed?

Conceding, however, to the Committee the merit of sincerity, the three individuals who manifested so much interest in this report, as to lead to the supposition that they would pay a part of the expense of publication if called on, must be regarded as the true culprits. One of this worthy triumvirate, Dr. Flint, was, it will be recollected, at one time a member of the Faculty, and of course had "authentic information" on all the matters involved. No man, therefore, knew better than he did the utter falsity of many if not all of the charges contained in this report. The Board of Managers, according to this document, is illegally constituted. Yet Dr. Flint accepted office under this same body, and most reluctantly yielded up his professorial privileges to it. As a member of the Faculty he was well aware that the expenditures during the first three years far exceeded the amount of matriculation fees, and actually joined in obtaining a considerable loan to supply the deficiency. Yet he has not hesitated to give currency to a paper charging the Faculty with having appropriated to their own uses the money thus derived from matriculations. By what code of ethics this gentleman's conduct is governed, it is difficult to imagine. Surely not by that which obtains among honest and upright men. What apology he may frame for himself out of the circumstances attending his ejection from the Faculty of the Institute at a former period, we are unable to say. We can conceive of nothing, however, that would justify him in pursuing a course of conduct such as is exposed by this pamphlet, and which must be looked upon not merely as disingenuous but disreputable. C.

